# Portal vein leiomyosarcoma: a case report and review of the literature

**DOI:** 10.1186/s12893-016-0174-x

**Published:** 2016-09-01

**Authors:** Antje E. Gohrbandt, Torsten Hansen, Christian Ell, Stefan S. Heinrich, Hauke Lang

**Affiliations:** 1 Department of General, Visceral and Transplantation Surgery, University Hospital of the Johannes Gutenberg University Mainz, Mainz, Germany; 2 Department of Pathology, University Hospital of the Johannes Gutenberg University Mainz, Mainz, Germany; 3 Department of Internal Medicine II/IV, Sana Klinikum, Offenbach, Germany; 4 Department of General, Visceral and Transplantation Surgery, University Hospital of Mainz, Mainz, Germany

**Keywords:** Leiomyosarcoma, Portal vein, Surgery, Review

## Abstract

**Background:**

Leiomyosarcoma of vascular smooth muscle is a very rare entity. A fair number of cases of vascular leiomyosarcomas have been reported, and the vast majority of these tumors arose from the inferior vena cava.

**Case Presentation:**

We report the case of a 71-year-old female patient who presented with recurrent upper abdominal pain. A CT-scan demonstrated a heterogenous mass in the liver hilum. Liver function tests and hematology parameters as well as the tumor markers were normal. Due to the unclear diagnosis a percutaneous biopsy of this mass was performed and revealed leiomyosarcoma. The patient was treated by a right sided hemihepatectomy with portal vein reconstruction and an end-to-side hepatico-jejunostomy. Final histology confirmed complete (R0) resection of a moderately differentiated leiomyosarcoma of the portal vein. After complete (R0) resection of the lesion, the patient remained without any signs of tumor recurrence for a total of 36 months until detection of an unresectable local recurrence. After surgical re-exploration the patient was finally referred to palliative radiotherapy.

**Conclusion:**

Vascular leiomyosarcoma of the portal vein is an extremely rare tumor entity. We have described a case with no evidence-based neo/adjuvant treatment options, where aggressive surgery achieved a tumor-free margin (R0), performed in a specialized center for sarcoma and hepatobiliary surgery.

## Background

Leiomyosarcoma is a malignant mesenchymal tumor of smooth muscle cells representing the second most frequent malignant soft tissue tumor in adults after liposarcoma. Perl initially described vascular leiomyosarcoma in 1871, which is a rare disease: so far, only a few hundred patients with vascular leiomyosarcoma have been reported in the literature. Vascular leiomyosarcoma most often arise from the inferior vena cava (IVC). Less frequently they have been described in the renal, mesenteric, hepatic and saphenous veins, or even in arteries [1–5]. Only one leiomyosarcoma of the portal vein has been described in the English or German literature so far [6].

## Case Presentation

**Fig. 1:**
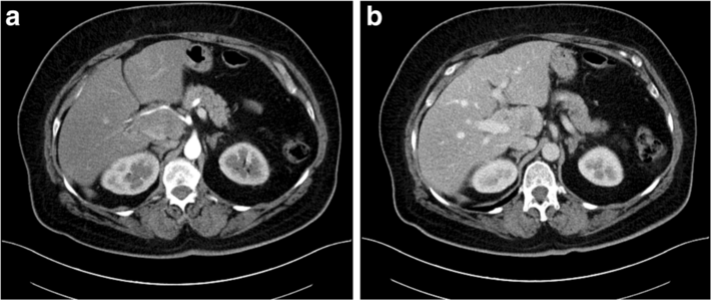
The preoperative contrast enhanced computed tomography scan demonstrated a heterogenous mass in the liver hilum which infiltrated the right portal vein. The hepatic artery was displaced by the tumor. However, CT did not reveal infiltration of the hepatic artery

We report the case of a 71-year-old female patient who presented with recurrent upper abdominal pain. CT-scan demonstrated a heterogenous mass in the liver hilum, which infiltrated the main portal vein up to the right branch. The common hepatic artery was displaced to the left but not encased by the tumor. No signs of biliary obstruction were seen (Fig. 1).

Liver function tests and hematology parameters as well as the tumor markers α-fetoprotein, CA 19–9, CEA, and chromogranin A were normal. Due to the unclear diagnosis, a percutaneous biopsy was performed which revealed leiomyosarcoma. Since no distant metastases were evident upon staging CT, the patient was referred to our centre for surgical treatment.

**Fig. 2:**
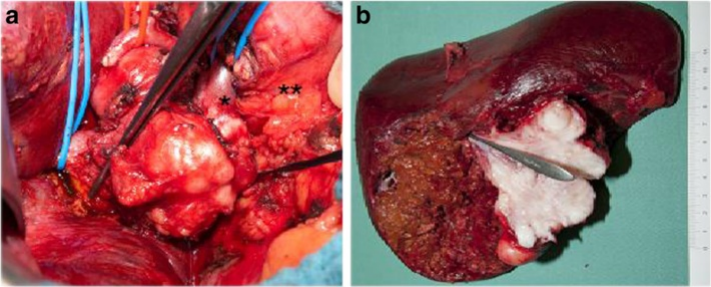
Intraoperative situs prior to resection (**a**): the portal vein was involved by the tumor from the upper margin of the pancreas up to the liver on the right side. The left portal vein was marked with *blue rubber bands* – both, the left branch (*) and supra-pancreatic part (**) were tumor-free. The right portal vein is marked by the forceps. The bile duct has been resected. The right hepatic artery has been ligated and the common hepatic artery has been fully mobilized to the left (*red rubber band*). In the resected specimen (**b**), the tumor has been split and the orifice of the right portal vein is intubated

In February 2012, surgical exploration was performed, which confirmed the extension of the tumor from the main portal vein into the right portal branch. In contrast to the preoperative staging, the tumor infiltrated both the right hepatic artery and the bile duct. The left branches of the hepatic artery, portal vein and bile duct were not affected by the tumor (Fig. 2a).

Due to the extent of the tumor, a right hepatectomy was performed with an en-bloc resection of the hilar bile duct bifurcation and lymphadenectomy. Reconstruction of the portal vein was achieved by an end-to-end-anastomosis, and an end-to-side hepatico-jejunostomy was performed for biliary reconstruction (Fig. 2b).

Postoperatively, the liver function was normal, and serial duplex ultrasound examinations and CT-scans confirmed regular flow in the portal vein. A bile leak from the hepatico-jejunostomy caused an abscess in the liver hilum and required an additional percutaneous drainage and antibiotic therapy. The patient was discharged from hospital 6 weeks postoperatively in good general condition.

**Fig. 3:**
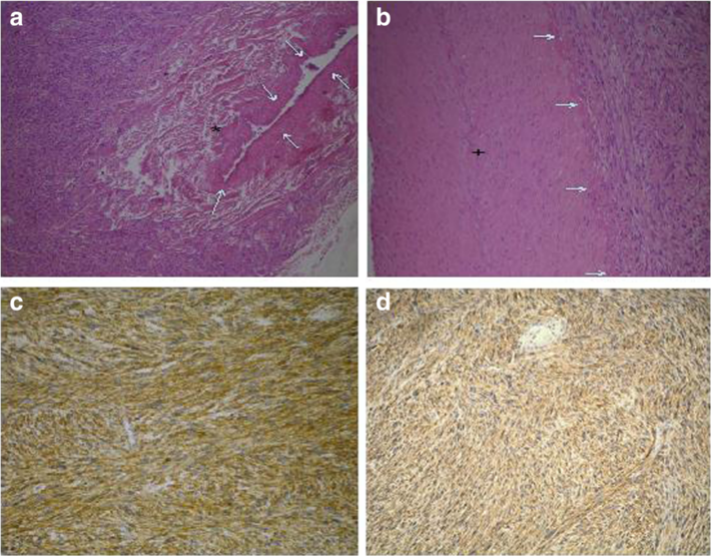
**a** Hematoxylin-eosin (HE) staining of the surgical specimen (magnification × 40). Arrows surrounding the portal vein, bundles of neoplastic smooth muscle cells partially infiltrating the portal vein (*). **b** HE staining of the surgical specimen (magnification × 100). Arrows indicate the adventitia of the hepatic artery without infiltration of the arterial wall (+). **c**, **d** The tumor stained positive for smooth muscle actin (**c**) and caldesmon (**d**) upon immunohistochemistry (magnification × 100)

Based on the microscopic morphology and immunhistochemic detection of strong expression of Actin and Caldesmon within the tumor cells the diagnosis of a moderately differentiated leiomyosarcoma (G2) with up to 15 mitoses in 10 high power fields was made. Necrotic areas were only found in about 5 % of the tumor volume (FNCLCC-score: 5/8). For the definition of resection status, the specimen was microscopically evaluated for the resection status by sampling of the distal and proximal resection margins of the portal vein, bile duct, right hepatic artery as well as the parenchymal resection margin of the liver. Complete resection (R0) was confirmed (Fig. 3).

**Fig. 4:**
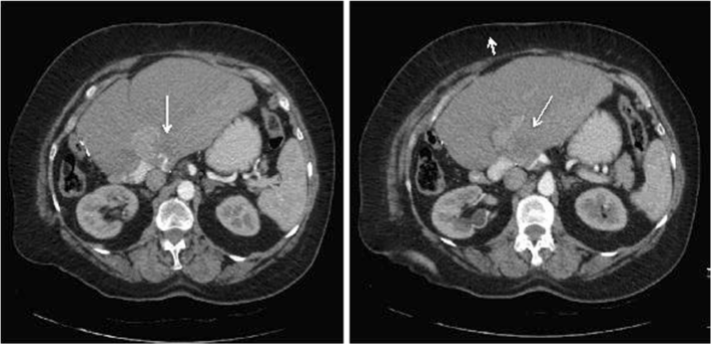
The CT scan 36 months after curative resection reveals intrahepatic tumor recurrence (*arrows*) arising from the left portal vein. The heterogenous texture of the liver on the right side of the tumor is caused by heterogenous perfusion of this hepatic area and is constant since primary surgery

The patient did not receive adjuvant treatment. A follow-up CT-scan 36 months postoperatively revealed tumor recurrence in the liver hilum (Fig. 4). Due an excellent general condition, the patient underwent re-exploration, which unfortunately revealed an unresectable local recurrence in the portal vein. The patient recovered quickly from this re-operation and received palliative radiotherapy with 50.4Gy until June 2015. The first follow-up MR imaging demonstrated a partial response of the tumor.

## Discussion

Vacular leiomyosarcoma is a rare tumor entity, which predominantly occurs in the IVC: more than 250 patients have been entered into a patient registry for this disease [4, 7]. An analysis of this registry indicated surgical resection of the tumor with negative margins as the most important prognostic factor, which achieves a 5-year survival of 33–68 % [8]. In contrast, only one case of leiomyosarcoma from the portal vein has been reported in the literature, yet. This tumor was however unresectable, and no long-term follow-up was reported from this case [6]. More recently, Frazzoni and colleagues described a leiomyosarcoma in a 44-year old female near the portal vein without infiltration of the vascular wall. Tumor recurrence was found 27 month after curative resection, and the patient died 47 months after surgery [9]. Similarly, our patient developed local recurrence after 36 months. Due to the limited follow-up after radiotherapy, the long-term survival is unknown.

In addition to the rare incidence, the variable histological appearance may complicate the diagnosis of vascular leiomyosarcoma. Due to the etiology of leiomyosarcoma, it usually shares characteristics of benign leiomyoma such as bundles of smooth muscle cells with central cigar-shaped nucleus, but cells also have eosinophilic cytoplasm and often perinuclear vacuoles. Usually, leiomyosarcoma stains positive for vimentin and smooth muscle actin, and they typically express desmin. Commonly, these tumors are negative for protein S-100 and neuron-specific enolase. However, expression of protein S-100, Leu 7, and epithelial membrane antigen have also been reported for leiomyosarcoma [2]. Morphology and immunohistochemical staining of the present case are in line with these features and thus confirm the diagnosis of leiomyosarcoma (Fig. 3).

Also due to the low incidence of this tumor, no adjuvant or neoadjuvant chemotherapy or radiotherapy regimen has been established for vascular leiomyosarcoma [2, 4, 7]. Up to date, radical resection aiming at complete local tumor clearance remains the primary treatment modality for patients with resectable vascular leiomyosarcoma and may achieve a favourable outcome [2–4, 7, 8, 10]. Considering the local recurrence in our patient, an adjuvant radiation therapy should have been considered. However, an adjuvant treatment is not recommended in the literature. Furthermore, the patient was discharged from hospital six weeks after surgery following a complicated course with an infectious complication. Therefore, the patient did not receive adjuvant therapy based on an interdisciplinary board discussion. Moreover, a de-novo sarcoma of a portal venous branch three years after the initial resection should also be taken into consideration, since the development of a real local recurrence with such delay after an apparently R0-resection appears unusal. Also, down-sizing therapies for unresectable tumors have failed to improve prognosis in a limited number of patients [8].

## Conclusions

Vascular leiomyosarcoma of the portal vein is an extremely rare tumor entity. In the absence of established neo/adjuvant treatment options, aggressive surgery seems to be the most reasonable treatment if tumor-free margins (R0) can be achieved. Based on the complexity of the hilar anatomy and the prognosis of unresectable disease, the treatment of this tumor entity should only be performed in specialized centres for sarcoma and hepato-biliary surgery.

## Abbreviations

CT: Computertomography
IVC: Inferior vena cava
CEA: Carcinoembryonic antigen
Ca 19–9: Carbohydrate antigen 19–9
FNCLCC: Fèdèration Nationale des Centres de Lutte le Cancer
